# RETRACTION: Downregulation of the Proton-Activated Cl- Channel TMEM206 Inhibits Malignant Properties of Human Osteosarcoma Cells

**DOI:** 10.1155/omcl/9816894

**Published:** 2025-11-20

**Authors:** Oxidative Medicine and Cellular Longevity

RETRACTION: F. Peng, H. Li, J. Li, and Z. Wang, “Downregulation of the Proton-Activated Cl- Channel TMEM206 Inhibits Malignant Properties of Human Osteosarcoma Cells,” *Oxidative Medicine and Cellular Longevity*, 2021, 3672112, https://doi.org/10.1155/2021/3672112.

The above article, published online on 05 November 2021 in Wiley Online Library (wileyonlinelibrary.com), has been retracted by agreement between the journal Editor-in-Chief, Jeannette Vasquez-Vivar, and John Wiley & Sons Ltd. The retraction has been agreed due to multiple concerns with the figures, also raised on PubPeer [1]. Specifically,

Figure 1a

• The GAPDH bands appear similar when flipped horizontally to the GAPDH bands shown in Figure 3a

Figure 1b

• Normal TMEM206 panel appears identical to the:
  ⚬ Para-tumor tissue panel of Figure 1e in [2]
  ⚬ Normal panel of Figure 2a in [3] (rotated 180 degrees)
• Stage I and Stage IIA panels appear identical to UICC Stage I and UICC Stage II panels of Figure 1e in [2]
• Stage IIB and Stage III panels appear identical to:
  ⚬ UICC Stage III and UICC Stage IV panels of Figure 1e in [2]
  ⚬ Primary and Recurrent panels of Figure 2a in [3]

Figure 2a

• All panels appear identical Figure 2a of [4]
• All panels appear identical to Figure 6 of [5]

Figure 2c

• U2OS panels appear identical to the DU145 panels shown in Figure 6a in [6]
• MG63 panels appear identical to the PC3 panels shown in Figure 6b in [6]

Figure 2e

• U2OS, SC-shRNA panel appears identical to 5637, RBMX−/hnRNP A1+ panel in Figure 4C of [7]
• U2OS, TMEM206-shRNA1 panel appears identical to 5637, RBMX−/hnRNP A1− panel in Figure 4C of [7]
• U2OS, TMEM206-shRNA2 panel appears identical to 5637, RBMX+/hnRNP A1− panel in Figure 4C of [7]
• MG63, SC-shRNA panel appears identical to 5637, RBMX−/PKM2+ panel in Figure 7C of [7]
• MG63, TMEM206-shRNA1 panel appears identical to 5637, RBMX+/PKM2+ panel in Figure 7C of [7]
• MG63, TMEM206-shRNA2 panel appears identical to 5637, RBMX−/PKM2− panel in Figure 7C of [7]

Figure 3b

• U2OS panels appear identical to the A549 panels shown in Figure 5a in [8]
• MG63 panels appear identical to the SK-MES-1 panels shown in Figure 5a in [8]

Figure 5a

• MG63/SC-shRNA panel identical to the pc-DNA-Con panel in [9]

Figure 7a

• Sc-shRNA panels appear identical to PBS panels in Figure 4a,b in [10]
• TMEM206-shRNA1 panels appear identical to MASP panels in Figure 4a,b in [10]
• TMEM206-shRNA2 panels appear identical to HASP panels in Figure 4a,b in [10]

Figure 7d

• TMEM206-shRNA1 panel appears identical to Ccr2−/− panel in bottom left of Figure 2b in [11]
• TMEM206-shRNA2 panel appears identical to Ccr2^ec^KO panel in top right of Figure 2b in [11]

The authors did not respond to the retraction.
